# Phase-type models for competing risks, with emphasis on identifiability issues

**DOI:** 10.1007/s10985-022-09547-7

**Published:** 2022-02-08

**Authors:** Bo Henry Lindqvist

**Affiliations:** Norwegian University of Science and Technology, Trondheim, Norway

**Keywords:** Phase-type distribution, Coxian distribution, Competing risks, Identifiability

## Abstract

We first review some main results for phase-type distributions, including a discussion of Coxian distributions and their canonical representations. We then consider the extension of phase-type modeling to cover competing risks. This extension involves the consideration of finite state Markov chains with more than one absorbing state, letting each absorbing state correspond to a particular risk. The non-uniqueness of Markov chain representations of phase-type distributions is well known. In the paper we study corresponding issues for the competing risks case with the aim of obtaining identifiable parameterizations. Statistical inference for the Coxian competing risks model is briefly discussed and some real data are analyzed for illustration.

## Introduction

A phase-type distribution can be defined to be the distribution of the time to absorption for an absorbing finite state Markov chain in continuous time. Initially, phase-type distributions received most attention in applied probability, in particular in queuing theory, generalizing the Erlang distribution. A much cited introduction to phase-type distributions is Neuts (1981). Important theoretical contributions are found in Cumani (1982) and a series of papers by O’Cinneide (e.g., O’Cinneide 1989). Aalen (1995) and (Aalen et al. 2008, Ch. 10) provide useful introductions aimed at applications in survival analysis.

There have recently appeared a number of articles involving the use of phase-type distributions in statistical modeling and inference. In particular there seems to be a particular interest in the use of so-called Coxian phase-type models, first suggested by Cox (1955). Their usefulness stems from the fact that they are able to model phenomena where the individuals go through stages (phases) in a specified order, and may transit to the absorbing state (corresponding to the event of interest) from any phase. Another reason for using Coxian phase-type models is that they apparently can be modeled by considerably fewer parameters than a general phase-type distribution. They are hence also better suited when covariates are included in the models. Coxian phase-type models have recently been successfully applied in health care studies. For example, Faddy et al. (2009), McGrory et al. (2009), Tang et al. (2012) and Rizk et al. (2021) model hospital length of stay by Coxian phase-type models.

The problem of fitting more general phase-type distributions to lifetime data has been considered in a frequentist setting using the EM-algorithm by Asmussen et al. (1996) and in a Bayesian setting using MCMC by Bladt and Gonzalez (2003), Aslett and Wilson (2011) and Laache (2014). Slud and Suntornchost (2014) advocated the use of parametric models based on phase-type distributions with a low number of parameters. One of their conclusions was that simple phase-type models can do almost as well as nonparametric methods in many lifetime studies.

The main purpose of the present paper is to study how the phase-type methodology can be modified to include competing risks, extending earlier work in this direction by Lindqvist (2013) and Lindqvist and Kjølen (2018). One then considers a finite state Markov chain with more than one absorbing state, each of which corresponds to a particular risk. The relevant observation will be the pair (*T*, *C*) where *T* is the time of absorption, while *C* denotes the absorbing state. This corresponds exactly to the observation of a classical competing risks model. Standard functions from the theory of competing risks (Lawless 2003, Ch. 9), (Aalen et al. 2008, Ch. 3) can now be given in terms of the transition matrix of the underlying Markov chain, see Sect. 3.1.

We will be particularly concerned with the uniqueness of parameterizations of phase-type models for competing risks. This will extend the study of Lindqvist and Kjølen (2018), who considered models with two transient states. It will also extend results from Cumani (1982), Telek and Horváth (2007) and Rizk et al. (2019) who considered uniqueness properties of parameterizations in the ordinary Coxian phase-type case.

Phase-type models with multiple absorbing states have earlier been considered by, e.g., McClean et al. (2010) and Rizk et al. (2021) in the modeling of patient pathways in hospitals. The former paper is concerned with the calculation of some key performance indicators like absorption probabilities and expected length of stay. The latter paper considers patient movements between a series of stations, each with the option of leaving the hospital. Their approach will be further considered in Sect. 3.4.

A large part of the present paper, particularly Sect. 2, concerns basic theoretical results and approaches on phase-type distributions that are essentially known and treated in several articles and books. They are needed here in order to explain their extensions to the competing risks case. For these purposes, it has also been necessary to handle different approaches and viewpoints in the literature. This applies to, for example, representations in terms of Laplace transforms involving *poles*, versus representations using transition matrices, involving *eigenvalues*.

The rest of this paper is organized as follows. In Sect. 2 we review basic results for ordinary phase-type distributions with a view towards uniqueness of their Markov chain representations. Representations for Coxian distributions are considered in particular. We also review briefly the basic functions of classical competing risks theory. In Sect. 3 we consider phase-type modeling for competing risks and show how results for the single absorbing state case are extended to multiple absorbing states. An example of fitting a Coxian competing risks model to real data is given. Some concluding remarks are given in Sect. 4. The paper is ended by an Appendix presenting technical derivations and discussions related to matrices, as well as the proof of one of the theorems.

## Background theory

We shall let vectors and matrices be given by bold letters, respectively lowercase and uppercase. Vectors will always be assumed to be column vectors. We shall use ${{\mathbf {I}}}$ to mean the identity matrix, where the dimension will be clear from the connection. This also applies to the use of the vectors ${{\mathbf {0}}}$ and ${{\mathbf {1}}}$ which are, respectively, vectors of all 0s and all 1s. Transposes of vectors will be marked by $'$, e.g. ${{\mathbf {p}}}'$. A vector of length *r* will for short be called an *r*-vector.

### Phase-type distributions

Phase-type distributions can be described in terms of a continuous time Markov process $\{ X(t); t \ge 0\}$, where the system moves through some or all of *m* transient states, or phases, before moving to a single absorbing state $m + 1$. The time of absorption, *T*, is then said to have a phase-type distribution.

The infinitesimal transition matrix ${{\mathbf {A}}}$ of the Markov chain that produces the phase-type distribution, is an $(m+1) \times (m+1)$ matrix given in block form as

1 $$\begin{aligned} {{\mathbf {A}}}= \left[ \begin{array}{cc} {{\mathbf {Q}}}&{} \quad {\varvec{\ell }}\\ {{\mathbf {0}}}' &{} \quad 0 \end{array} \right] . \end{aligned}$$

Here ${{\mathbf {Q}}}$ is the $m \times m$ matrix corresponding to transitions between the transient states; ${\varvec{\ell }}$ is the *m*-vector defining direct transition intensities from the transient states to the absorbing state. Letting ${{\mathbf {P}}}(t)$ be the matrix of transition probabilities $P_{ij}(t)=P(X(t)=j|X(0)=i)$ it is well known (e.g., Ross 2010, Chapter 5) that

$$\begin{aligned} {{\mathbf {P}}}(t) = e^{{{\mathbf {A}}}t} = \sum _{i=0}^\infty \frac{t^i}{i!}{{\mathbf {A}}}^i, \end{aligned}$$

and it is then straightforward to show that (1) implies

2 $$\begin{aligned} {{\mathbf {P}}}(t) = \left[ \begin{array}{cc} e^{{{\mathbf {Q}}}t} &{} \quad {{\mathbf {Q}}}^{-1}(e^{{{\mathbf {Q}}}t} - {{\mathbf {I}}}){\varvec{\ell }}\\ {{\mathbf {0}}}' &{} \quad 1 \end{array} \right] . \end{aligned}$$

Now let ${{\mathbf {p}}}$ be the *m*-vector with entries $p_i = P(X(0)=i)$ for $i=1,\ldots ,m$, where $\sum _{i=1}^m p_i = 1$. Thus ${{\mathbf {p}}}$ defines the initial distribution of the Markov chain, assuming that the chain starts in a transient state.

Note that the transition matrix ${{\mathbf {A}}}$ is completely determined by ${{\mathbf {Q}}}$. This is because all row sums of ${{\mathbf {A}}}$ are 0 so that ${\varvec{\ell }}= -{{\mathbf {Q}}}{{\mathbf {1}}}$. Hence a phase type distribution is determined by the pair $({{\mathbf {p}}},{{\mathbf {Q}}})$.

By using (2) we are able to express the standard functions describing the probability distribution of *T* in terms of the given matrix representation. The cumulative distribution function of *T* is

$$\begin{aligned} F(t)= P(T \le t) = P(X(t)=m+1) = {{\mathbf {p}}}' {{\mathbf {Q}}}^{-1}(e^{{{\mathbf {Q}}}t} - {{\mathbf {I}}}){\varvec{\ell }}. \end{aligned}$$

Taking the derivative with respect to *t* we get the density function

3 $$\begin{aligned} f(t)= {{\mathbf {p}}}' e^{{{\mathbf {Q}}}t} {\varvec{\ell }}. \end{aligned}$$

The survival function *S*(*t*) can be found from $S(t)=1-F(t)$, or by noting that

4 $$\begin{aligned} S(t)= P(T > t) = P(X(t) \in \{1,2,\ldots ,m\}) = {{\mathbf {p}}}' e^{{{\mathbf {Q}}}t}{{\mathbf {1}}}. \end{aligned}$$

This leads in turn to the following expression for the hazard function of *T*,

5 $$\begin{aligned} \lambda (t)= \frac{{{\mathbf {p}}}' e^{{{\mathbf {Q}}}t} {\varvec{\ell }}}{{{\mathbf {p}}}' e^{{{\mathbf {Q}}}t} {{\mathbf {1}}}}. \end{aligned}$$

### Representations and identifiability

As is well known, representations $({{\mathbf {p}}},{{\mathbf {Q}}})$ of phase-type distributions are not unique. The representation $({{\mathbf {p}}},{{\mathbf {Q}}})$ where ${{\mathbf {Q}}}$ is an $m \times m$ matrix, is said to be of *dimension* *m*. Following standard notation, the *order* of a phase-type distribution is the minimal dimension of all its representations.

The unique way of representing a phase-type distribution is through its Laplace transform, $f^*(s)=E(e^{-sT})$. It was shown by O’Cinneide (1990) that a distribution on the non-negative real numbers which is not the point mass at zero is a phase-type distribution if and only if (a) it has a strictly positive continuous density on the positive reals, (b) it has a rational Laplace transform with a unique pole of maximal real part.

For a given representation $({{\mathbf {p}}},{{\mathbf {Q}}})$, the Laplace transform can be written as

6 $$\begin{aligned} f^*(s) = {{\mathbf {p}}}' (s{{\mathbf {I}}}-{{\mathbf {Q}}})^{-1}(-{{\mathbf {Q}}}){{\mathbf {1}}}\end{aligned}$$

which is hence a rational function of *s*, i.e., of the form $f^*(s)=N(s)/D(s)$ for polynomials *N*(*s*) and *D*(*s*) (see Example 1 below for a simple case). The degree of the denominator polynomial, *D*(*s*), after having canceled possible equal factors in the numerator and denominator, is called the *degree* of the phase-type distribution. It was shown by O’Cinneide (1990) that the order of a phase-type distribution is at least as large as its degree, *but that it may be strictly larger.*

By the non-uniqueness of representations of phase-type distributions, it is of interest to consider the identifiability problem for these distributions, i.e., the problem of determining whether two representations $({{\mathbf {p}}}^{(a)},{{\mathbf {Q}}}^{(a)})$ and $({{\mathbf {p}}}^{(b)},{{\mathbf {Q}}}^{(b)})$ lead to the same phase-type distribution in the sense of having the same Laplace transform. In general, the problem of identifying representations $({{\mathbf {p}}},{{\mathbf {Q}}})$ for a given Laplace transform is a difficult one, where general recipes are still lacking. We refer to the concluding remarks in Sect. 4 for a further discussion of this.

We present below a theorem given by Telek and Horváth (2007) which compares two representations $({{\mathbf {p}}}^{(a)},{{\mathbf {Q}}}^{(a)})$ and $({{\mathbf {p}}}^{(b)},{{\mathbf {Q}}}^{(b)})$ of the same dimension. Following Telek and Horváth (2007), we shall say that a representation $({{\mathbf {p}}},{{\mathbf {Q}}})$ of dimension *m* is *nonredundant* if the degree of the corresponding phase-type distribution is *m*. For intuition, we may think of nonredundancy as a way of excluding representations of phase-type distributions where there are also representations with a lower dimension. Some simple illustrations are given in Example 1 below.

**Fig. 1:**
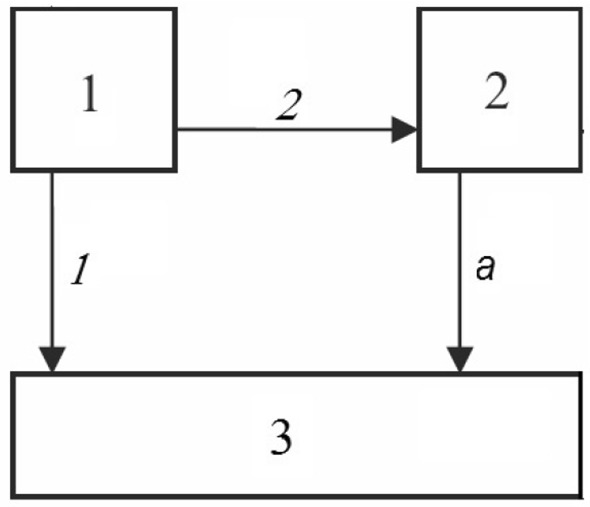
The phase-type model of Example 1

#### Example 1

(*Redundant phase-type distributions*) Let $m=2$ and consider the representation $({{\mathbf {p}}},{{\mathbf {Q}}})$ given by

7 $$\begin{aligned} {{\mathbf {p}}}' = (p,1-p), \; \; \; {{\mathbf {Q}}}= \left( \begin{array}{cc} -3 &{} \quad 2 \\ 0 &{} \quad -a \end{array} \right) , \end{aligned}$$

where $0 \le p \le 1$ and $a>0$. Figure 1 illustrates the corresponding Markov chain. A calculation using (6) leads to

8 $$\begin{aligned} f^*(s) =\left( a(1-p)+p\right) \frac{\left( s+\frac{3a}{a(1-p)+p}\right) }{(s+3)(s+a)}. \end{aligned}$$

Putting $a=1$, this equals $1/(s+1)$ which implies that $({{\mathbf {p}}},{{\mathbf {Q}}})$ is redundant for any value of *p*, with *T* having the standard exponential distribution. This may in fact be concluded directly from Fig. 1 by letting $a=1$ and observing that the transition rate to the absorbing state is 1 from each transient state.

Letting instead $a=5$, it can be seen that $({{\mathbf {p}}},{{\mathbf {Q}}})$ is redundant for $p=1/2$ and $p=0$. The resulting Laplace transforms are then, respectively, $1/(s+3)$ and $1/(s+5)$, corresponding to exponential distributions with respective rates 3 and 5.

#### Theorem 1

(Telek and Horváth 2007) Let $({{\mathbf {p}}}^{(a)},{{\mathbf {Q}}}^{(a)})$ and $({{\mathbf {p}}}^{(b)},{{\mathbf {Q}}}^{(b)})$ represent two nonredundant phase-type distributions with the same dimension *m*. Let their cumulative distribution functions be $F^{(a)}(t)$ and $F^{(b)}(t)$, respectively. Then $F^{(a)}(t)=F^{(b)}(t)$ for all *t* if and only if there exists a nonsingular $m \times m$ matrix ${{\mathbf {B}}}$ with ${{\mathbf {B}}}{{\mathbf {1}}}= {{\mathbf {1}}}$, such that ${{{\mathbf {p}}}^{(b)}}' = {{{\mathbf {p}}}^{(a)}}'{{\mathbf {B}}}$ and ${{\mathbf {Q}}}^{(b)} = {{\mathbf {B}}}^{-1} {{\mathbf {Q}}}^{(a)}{{\mathbf {B}}}$.

For the proof, we refer to Telek and Horváth (2007). A further discussion of the result and the assumption of nonredundancy is given in Appendix A2. As a corollary to the result, Telek and Horváth (2007) stated the following result, which is also well known from the literature, e.g., O’Cinneide (1999).

#### Theorem 2

(Telek and Horváth 2007) A general nonredundant phase-type distribution of order *m* can be fitted by $2m-1$ independent parameters.

This result is indeed of special interest for phase-type modeling, since apparently a representation $({{\mathbf {p}}},{{\mathbf {Q}}})$ would need $m^2+m-1$ independent parameters.

The following example illustrates the result of Theorem 1. It shows in particular that ${{\mathbf {B}}}$ need not be nonnegative.

#### Example 2

(Equivalent nonredundant phase-type distribution) Let ${{\mathbf {Q}}}^{(a)}$ be the matrix in (7) with $a=5$ and let a $2 \times 2$-matrix ${{\mathbf {B}}}$ be given by

9 $$\begin{aligned} {{\mathbf {B}}}= \left( \begin{array}{rcr} 2 &{} \quad ~&{} \quad -1 \\ -1 &{} \quad ~&{} \quad 2 \end{array} \right) . \end{aligned}$$

This matrix is nonsingular with row sums 1, as required in Theorem 1. Let

$$\begin{aligned} {{\mathbf {Q}}}^{(b)} = {{\mathbf {B}}}^{-1}{{\mathbf {Q}}}^{(a)} {{\mathbf {B}}}= \left( \begin{array}{rcr} -11/3 &{} \quad ~ &{} \quad 4/3 \\ 2/3 &{} \quad ~&{} \quad -13/3 \end{array} \right) . \end{aligned}$$

Letting ${{{\mathbf {p}}}^{(a)}}' = (3/5,2/5)$ and ${{{\mathbf {p}}}^{(b)}}' ={{{\mathbf {p}}}^{(a)}}' {{\mathbf {B}}}= (4/5,1/5)$, it follows from Theorem 1 that $({{\mathbf {p}}}^{(a)},{{\mathbf {Q}}}^{(a)})$ and $({{\mathbf {p}}}^{(b)},{{\mathbf {Q}}}^{(b)})$ represent the same phase-type distribution.

### Triangular and Coxian phase-type distributions

#### Canonical representations of Coxian distributions

The class of *Coxian* phase-type distributions are defined via Markov chains with *m* transient states, where the possible transitions from a transient state *i* is either to state $i+1$ or to the absorbing state $m+1$. More specifically, a Coxian distribution can be represented by the pair $({{\mathbf {p}}},{{\mathbf {Q}}})$ with ${{\mathbf {p}}}=(1,0,\ldots ,0)^\prime$ and ${{\mathbf {Q}}}$ given on the form

10 $$\begin{aligned} {{\mathbf {Q}}}= \left( \begin{array}{ccccc} -\lambda _1 &{} \quad \alpha _{12} &{} \quad 0 &{} \quad \cdots &{} \quad 0 \\ 0 &{} \quad -\lambda _2 &{} \quad \alpha _{23} &{} \quad \cdots &{} \quad 0 \\ \vdots &{} \quad \vdots &{} \quad \vdots &{} \quad \ddots &{} \quad \vdots \\ 0 &{} \quad 0 &{} \quad 0 &{} \quad \cdots &{} \quad -\lambda _m \end{array} \right) \end{aligned}$$

It is seen that the Coxian distribution is then represented by $2m-1$ parameters (see Theorem 2). Fig. 1 illustrates the special case $m=2$.

Coxian phase-type distributions are, however, more general than they might look. In fact, O’Cinneide (1990) proved the remarkable result that any probability measure on $(0,\infty )$ with rational Laplace transform with *only real poles*, and with a continuous positive density on $(0, \infty )$, is a Coxian distribution.

**Fig. 2:**
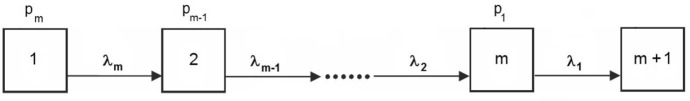
The path of the canonical representation for an upper triangular distribution using $({{\tilde{{{\mathbf {p}}}}}},{{\tilde{{{\mathbf {Q}}}}}})$

The matrix ${{\mathbf {Q}}}$ in (10) is in particular upper triangular. The subfamily of phase-type distributions with upper triangular ${{\mathbf {Q}}}$ are of special interest in applications, since they represent non-cyclic Markov chains *X*(*t*). A special treatment of these distributions is found in the much cited paper Cumani (1982) (see also Bobbio and Cumani 1992 for a review of the main results). Working with Laplace transforms, Cumani (1982) proved that any phase-type distribution with an upper triangular ${{\mathbf {Q}}}$ can be represented uniquely in the form $({{\tilde{{{\mathbf {p}}}}}},{{\tilde{{{\mathbf {Q}}}}}})$ where ${{\tilde{{{\mathbf {Q}}}}}}$ is given by

11 $$\begin{aligned} {{\tilde{{{\mathbf {Q}}}}}} = \left( \begin{array}{ccccc} -\lambda _m &{} \quad \lambda _m &{} \quad 0 &{} \quad \cdots &{} \quad 0 \\ 0 &{} \quad -\lambda _{m-1} &{} \quad \lambda _{m-1} &{} \quad \cdots &{} \quad 0 \\ \vdots &{} \quad \vdots &{} \quad \vdots &{} \quad \ddots &{} \quad \vdots \\ 0 &{} \quad 0 &{} \quad 0 &{} \quad \cdots &{} \quad -\lambda _1 \end{array} \right) \end{aligned}$$

with $\lambda _1\ge \lambda _2\ge \ldots ,\ge \lambda _m$, and where ${{\tilde{{{\mathbf {p}}}}}} = (p_m,p_{m-1},\ldots ,p_1)'$ is a probability vector. Figure 2 illustrates the representation. Observe that there are $2m-1$ free parameters.

The representation $({{\tilde{{{\mathbf {p}}}}}},{{\tilde{{{\mathbf {Q}}}}}})$ suggests a way of calculating the Laplace transform for the absorption time *T*. The distribution of *T* given $X(0)=m-i+1$ is now a convolution of *i* independent exponential variables with rates $\lambda _1,\lambda _{2},\ldots ,\lambda _i$ (see Fig. 2). It hence follows that the Laplace transform of *T* is

12 $$\begin{aligned} f^*(s)=\sum _{i=1}^m p_i g^*_{(i)}(s), \end{aligned}$$

where $$p_i=P(X(0)=m-i+1)$$ and

13 $$\begin{aligned} g^*_{(i)}(s)= \frac{\lambda _1 \lambda _{2} \cdots \lambda _i}{ (s+\lambda _1)(s+\lambda _{2}) \cdots (s+\lambda _i)} \end{aligned}$$

is the Laplace transform of the appropriate convolution. We may hence write $f^*(s)$ in the form *N*(*s*)/*D*(*s*), where $D(s)=\prod _{i=1}^m(s+\lambda _i)$, which is the general form considered in Sect. 2.2.

Consider now an arbitrary phase-type distribution with a given upper triangular ${{\mathbf {Q}}}$ and Laplace transform ${{\hat{N}}}(s)/{{\hat{D}}}(s)$, say. As demonstrated by Cumani (1982), this model may be put in the above canonical form by letting $D(s)={{\hat{D}}}(s)$ and deriving the $p_i$ in (12) by equating coefficients in the identity $\sum _{i=1}^m p_ig^*_{(i)}(s) = {{\hat{N}}}(s)/{{\hat{D}}}(s)$. Cumani (1982) noted that the resulting set of equations for the $p_i$ can be put in triangular form and is hence easy to solve and gives a unique solution with $p_i \ge 0$ and $\sum _ip_i=1$. The example below gives a simple illustration.

##### Example 3

Consider the representation $({{\mathbf {p}}},{{\mathbf {Q}}})$ in Example 1 with $a=5,p=1$. The resulting Laplace transform is by (8),

$$\begin{aligned} f^*(s) = \frac{s+15}{(s+3)(s+5)}. \end{aligned}$$

We now want to put this in canonical form $({{\tilde{{{\mathbf {p}}}}}},{{\tilde{{{\mathbf {Q}}}}}})$. Then $\lambda _1=5,\lambda _2=3$ and hence

$$\begin{aligned} g^*_{(1)}(s) = \frac{5}{s+5}, \; \; \; g^*_{(2)}(s) = \frac{15}{(s+5)(s+3)}. \end{aligned}$$

We finally need to find $p_1,p_2$ so that (12) holds. A straightforward calculation gives $p_1=1/5,\; p_2=4/5$.

The Coxian distributions as defined by (10) and having initial vector ${{\mathbf {p}}}=(1,0,\ldots ,0)$ are apparently still of main interest for statistical modeling and inference, see for example the references given in the Introduction. Actually, the representation using (10) with ${{\mathbf {p}}}=(1,0,\ldots ,0)$ and assuming the ordering $\lambda _1 \ge \lambda _2 \ge \ldots \ge \lambda _m$, is presented by Cumani (1982) as an equivalent canonical version for upper triangular distributions, having a one-to-one explicit connection with the representation $({{\tilde{{{\mathbf {p}}}}}},{{\tilde{{{\mathbf {Q}}}}}})$. Note that the ordering of the $\lambda _i$ on the diagonal are opposite in the two matrices ${{\mathbf {Q}}}$ and ${{\tilde{{{\mathbf {Q}}}}}}$.

*Example 3 (cont.)* The canonical Coxian representation of the distribution in the first part of this example is

14 $$\begin{aligned} {{\mathbf {Q}}}= \left( \begin{array}{rrr} -5 &{} \quad ~&{} \quad 4 \\ 0 &{} \quad ~&{} \quad -3 \end{array} \right) , \end{aligned}$$

The representation (14) is hence equivalent to the representation from (7), where the diagonal elements are interchanged.

Apparently, the Coxian representation (10) is well defined also when the eigenvalues $\lambda _i$ on the diagonal are ordered differently from the decreasing order. This is true, but it turns out that the set of phase-type distributions represented by the matrix (10), with any other predetermined order of the diagonal elements, may not contain all upper triangular phase-type distributions (see remark at the end of Example 4 below). This fact is inherent in Cumani (1982). Equivalently, the canonical representation of Fig. 2 requires an increasing order of the $\lambda _i$ from left to right.

#### Redundancy of Coxian distributions

Consider a Coxian distribution given in canonical form using (10). It is clear that if $\alpha _{k,k+1}=0$ for some $k \in \{1,2,\ldots ,m-1\}$, then this representation is redundant. In fact, the distribution can be represented by a matrix in the form (10) of dimension *k*. The above is, moreover, equivalent to having $p_{k+1}=p_{k+2}=\ldots =p_{m}=0$ in the representation using ${{\tilde{{{\mathbf {Q}}}}}}$. As seen from the following example due to O’Cinneide (1989), the possible redundancy of Coxian phase-type distributions does, however, not always arise from cases where $p_j=0$ from some *j* on.

##### Example 4

(O’Cinneide 1989) Let the canonical representation $({{\tilde{{{\mathbf {p}}}}}},\tilde{{\mathbf {Q}}})$ be given by $m=4$, ${{\tilde{{{\mathbf {p}}}}}}=(1/2,0,0,1/2)^\prime$ and $\lambda _1=4,\lambda _2=3,\lambda _3=2,\lambda _4=1$. Hence $p_m \ne 0$, so we cannot use the above observation to conclude redundancy. A calculation of the Laplace transform shows, however, redundancy because a factor $(s+4)$ is common in numerator and denominator. O’Cinneide (1989) pointed to the remarkable fact that, despite the redundancy, there is still no Markov chain representation with dimension $m=3$ of the underlying phase-type distribution. This can be seen by solving for the $p_i$ in (12), where a negative value is found for $p_3$, hence implying no proper solution.

As an additional observation from this example, the underlying phase-type distribution cannot be represented by a Coxian representation ${{\mathbf {Q}}}$ where the ordering of the $\lambda _i$ is reversed.

#### Identifiability properties of Coxian phase-type distributions

The above shows that when using Coxian models one should be careful about the ordering of the $\lambda _i$ on the main diagonal of (10). Knowing the $\lambda _i$, there is always a valid and unique version of (10) with the $\lambda _i$ in decreasing order. If the $\lambda _i$ are put in another order, then valid representations may still be found, for example using Theorem 1. As noted above, however, changing the order of the $\lambda _i$ on the diagonal may lead to non-valid representations. Rizk et al. (2019) considered this problem and presented an algorithm that for any matrix of the form (10) finds all permutations of the diagonal entries that lead to valid representations in Coxian form. From Cumani (1982) follows that there is always at least one such representation, which however may be the only one as shown by an example in Rizk et al. (2019).

The algorithm of Rizk et al. (2019) is based on their Theorem 2, which we restate in the following theorem. This theorem gives an often useful condition for identifiability of Coxian phase-type models.

##### Theorem 3

(Rizk et al. 2019) Let ${{\mathbf {Q}}}^{(a)}$ and ${{\mathbf {Q}}}^{(b)}$ of same dimension *m* represent nonredundant Coxian phase-type distributions as given by (10), with diagonal elements ordered in the same way and with initial distributions ${{\mathbf {p}}}^{(a)}={{\mathbf {p}}}^{(b)} = (1,0,\ldots ,0)^\prime$. Let the corresponding cumulative distribution functions be $F^{(a)}(t)$ and $F^{(b)}(t)$, respectively. Then if $F^{(a)}(t)=F^{(b)}(t)$ for all *t*, we have ${{\mathbf {Q}}}^{(a)} = {{\mathbf {Q}}}^{(b)}$

### Classical competing risks

In competing risks, one observes the time to an event, *T*, in addition to the cause $C \in \{1,2,\ldots ,K\}$ for a positive integer *K*. The observation is hence a pair (*T*, *C*), where the joint distribution is completely specified by the subdistribution functions and the subdensities, given by respectively,

$$\begin{aligned} F_j(t) = P(T \le t, C=j), \; \; \; f_j(t) = F_j'(t) \end{aligned}$$

for $j=1,2,\ldots ,K$. The interpretation of $F_j(t)$ is as the probability of failing from cause *j* before time *t*. The $F_j(t)$ are also called *cumulative incidence functions*.

As an extension of the concept of hazard function of a lifetime distribution, one considers the *cause-specific hazard functions*,

$$\begin{aligned} \lambda _j(t) = \lim _{\varDelta t \rightarrow 0} \frac{P \left( t < T \le t + \varDelta t, C=j\;|\;T > t\right) }{\varDelta t} = \frac{f_j(t)}{S(t)}, \end{aligned}$$

where $S(t)=P(T>t)$. The interpretation is that $\lambda _j(t)$ is the hazard rate from cause *j* conditional on survival up to time *t*.

## Phase-type models for competing risks

Consider the general setup of Sect. 2.1 where the Markov chain $\{ X(t); t \ge 0\}$ moves among *m* transient states before it is absorbed in state $m + 1$. Suppose now instead that there are $K > 1$ absorbing states, named $m+1,m+2,\ldots ,m+K$, say. Let *T* be the time of absorption in any one of the absorbing states, and let the cause *C* represent the state where absorption occurs, defining $C=j$ if $X(T) = m+j; \;j=1,2,\ldots ,K$. Then the pair (*T*, *C*) can be viewed as an observation from a classical competing risks case with possible causes $1,\ldots ,K$.

**Fig. 3:**
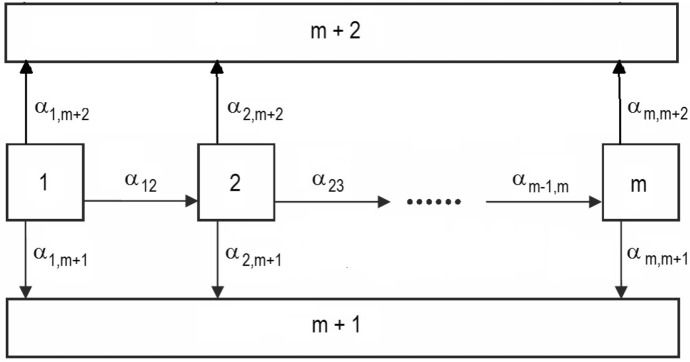
A Coxian phase-type model for $K=2$ competing risks

The ordinary Coxian phase-type model can now be extended to the competing risks case by allowing transitions to any of the *K* absorbing states $m+1,\ldots ,m+K$ from each of the transient states. The case $K=2$ is illustrated in Fig. 3.

### Representation of phase-type distributions for competing risks.

By extending the matrix (1) to include *K* absorbing states, we obtain the infinitesimal transition matrix of the modified Markov process to be the $(m+K) \times (m+K)$ matrix given in block form as

15 $$\begin{aligned} {{\mathbf {A}}}= \left[ \begin{array}{cc} {{\mathbf {Q}}}&{} \quad {{\mathbf {L}}}\\ {{\mathbf {0}}}_1 &{} \quad {{\mathbf {0}}}_2 \end{array} \right] . \end{aligned}$$

Here ${{\mathbf {Q}}}$ is the $m \times m$ matrix corresponding to transitions between the transient states, while the *m*-vector ${\varvec{\ell }}$ is replaced by the $m \times K$ matrix ${{\mathbf {L}}}$ of transition intensities from the transient states to the absorbing states. Further, ${{\mathbf {0}}}_1$ and ${{\mathbf {0}}}_2$ are, respectively, $K \times m$ and $K \times K$ matrices of zeros.

Similarly to the derivation of (2), we obtain the matrix of transition probabilities $P_{ij}(t)$ given by

16 $$\begin{aligned} {{\mathbf {P}}}(t) = \left[ \begin{array}{cc} e^{{{\mathbf {Q}}}t} &{} \quad {{\mathbf {Q}}}^{-1}(e^{{{\mathbf {Q}}}t} - {{\mathbf {I}}}){{\mathbf {L}}}\\ {{\mathbf {0}}}_1 &{} \quad {{\mathbf {I}}}\end{array} \right] . \end{aligned}$$

Let the *m*-vector ${{\mathbf {p}}}$ be the initial distribution of the Markov chain. The triple $({{\mathbf {p}}},{{\mathbf {Q}}},{{\mathbf {L}}})$ thus determines a competing risks distribution.

Observe that ${\varvec{\ell }}\equiv {{\mathbf {L}}}{{\mathbf {1}}}$ is the vector of transition rates from the transient states to the lumped set of absorbing states, and hence corresponds to the vector ${\varvec{\ell }}$ of the ordinary phase-type model considered in Sect. 2.1. It follows that ${{\mathbf {L}}}{{\mathbf {1}}}= - {{\mathbf {Q}}}{{\mathbf {1}}}$. Thus the $m\times K$ matrix ${{\mathbf {L}}}$ satisfies *m* conditions given by ${{\mathbf {Q}}}$ and has hence $Km-m=(K-1)m$ free parameters. Adding these to the $2m-1$ parameters of $({{\mathbf {p}}},{{\mathbf {Q}}})$, we have $(K+1)m-1$ parameters in the representation $({{\mathbf {p}}},{{\mathbf {Q}}},{{\mathbf {L}}})$.

From (16) we obtain expressions for the subdistribution functions, given by

$$\begin{aligned} F_j(t) = P(T \le t, C=j) = P(X(t)=m+j) = {{\mathbf {p}}}' {{\mathbf {Q}}}^{-1}(e^{{{\mathbf {Q}}}t} - {{\mathbf {I}}}){\varvec{\ell }}_j \end{aligned}$$

for $j=1,\ldots ,K$, where ${{\mathbf {p}}}$ is the *m*-vector defining the initial distribution of the Markov chain and ${\varvec{\ell }}_j$ is the *j*th column of ${{\mathbf {L}}}$. By differentiation we get the subdensities

17 $$\begin{aligned} f_j(t)=F_j'(t) ={{\mathbf {p}}}'e^{{\mathbf {Q}}t}{\varvec{\ell }}_j, \end{aligned}$$

while the cause-specific hazard rates are given by

18 $$\begin{aligned} \lambda _j(t)= \lim _{\varDelta t \rightarrow 0} \frac{P(T \le t+\varDelta t,C=j|T>t)}{\varDelta t} = \frac{f_j(t)}{S(t)} = \frac{{{\mathbf {p}}}' e^{{{\mathbf {Q}}}t} {\varvec{\ell }}_j}{{{\mathbf {p}}}' e^{{{\mathbf {Q}}}t} {{\mathbf {1}}}} . \end{aligned}$$

We shall below also need the Laplace transforms of the subdensities $f_j(t)$. Similarly to (6) we get

19 $$\begin{aligned} f_j^*(s) = {{\mathbf {p}}}' (s{{\mathbf {I}}}-{{\mathbf {Q}}})^{-1}{\varvec{\ell }}_j. \end{aligned}$$

### Identifiability of phase-type models for competing risks

Let $({{\mathbf {p}}},{{\mathbf {Q}}},{{\mathbf {L}}})$ be a phase-type model for competing risks. We shall call it *nonredundant* if $({{\mathbf {p}}},{{\mathbf {Q}}})$ is a nonredundant phase-type model as defined in Sect. 2.1.

The next theorem extends the result of Theorem 1 to the competing risks case. The proof is given in Appendix A3.

#### Theorem 4

Let $({{\mathbf {p}}}^{(a)},{{\mathbf {Q}}}^{(a)},{{\mathbf {L}}}^{(a)})$ and $({{\mathbf {p}}}^{(b)},{{\mathbf {Q}}}^{(b)},{{\mathbf {L}}}^{(b)})$ be two nonredundant phase-type representations for competing risks, having subdistribution functions $F_j^{(a)}(t)$ and $F_j^{(b)}(t)$, respectively. Then $F_j^{(a)}(t)=F_j^{(b)}(t)$ for all *t* and *j* if and only if there exists a nonsingular $m \times m$ matrix ${{\mathbf {B}}}$ with ${{\mathbf {B}}}{{\mathbf {1}}}= {{\mathbf {1}}}$ such that ${{{\mathbf {p}}}^{(b)}}' = {{{\mathbf {p}}}^{(a)}}'{{\mathbf {B}}}$, ${{\mathbf {Q}}}^{(b)} = {{\mathbf {B}}}^{-1} {{\mathbf {Q}}}^{(a)}{{\mathbf {B}}}$ and ${{\mathbf {L}}}^{(b)} = {{\mathbf {B}}}^{-1} {{\mathbf {L}}}^{(a)}$.

### Coxian phase-type models for competing risks

In the present subsection we specialize to the case of Coxian phase-type distributions for competing risks. These will be defined as the triple $({{\mathbf {p}}},{{\mathbf {Q}}},{{\mathbf {L}}})$ where ${{\mathbf {p}}}=(1,0,\ldots ,0)$, ${{\mathbf {Q}}}$ is given by (10), and ${{\mathbf {L}}}$ is an $m \times K$ matrix defined in the same way as in Sect. 3.1. Recalling that ${{\mathbf {Q}}}$ has $2m-1$ parameters, it follows from the reasoning of the cited subsection that the Coxian model has $(K+1)m-1$ parameters.

We can now prove the following result which extends Theorem 3 and is a simple consequence of Theorem 4. As for Theorem 3, we build upon the reasoning in Rizk et al. (2019).

#### Theorem 5

Consider two non-redundant Coxian phase-type distributions for competing risks, given by $({{\mathbf {p}}}^{(a)},{{\mathbf {Q}}}^{(a)},{{\mathbf {L}}}^{(a)})$ and $({{\mathbf {p}}}^{(b)},{{\mathbf {Q}}}^{(b)},{{\mathbf {L}}}^{(b)})$, where ${{\mathbf {p}}}^{(a)} = {{\mathbf {p}}}^{(b)}=(1,0,\ldots ,0)^\prime$. Assume further that the diagonals of ${{\mathbf {Q}}}^{(a)}$ and ${{\mathbf {Q}}}^{(b)}$ are ordered in the same way. Then if $F_j^{(a)}(t) = F_j^{(b)}(t)$ for all $t>0$ and $j=1,2,\ldots ,K$, we have

$$\begin{aligned} {{\mathbf {Q}}}^{(b)}= & {} {{\mathbf {Q}}}^{(a)} \\{{\mathbf {L}}}^{(b)}= & {} {{\mathbf {L}}}^{(a)} \end{aligned}$$

#### Proof

Let ${{\mathbf {B}}}$ be the invertible matrix obtained by using Theorem 4 for the present situation. Corollary 1 of Rizk et al. (2019) shows that if ${{\mathbf {p}}}^{(a)} = {{\mathbf {p}}}^{(b)}=(1,0,\ldots ,0)^\prime$, then the matrix ${{\mathbf {B}}}$ is lower triangular. Moreover, they show that if the ordering of the diagonal elements of ${{\mathbf {Q}}}^{(a)}$ and ${{\mathbf {Q}}}^{(b)}$ are the same, then ${{\mathbf {B}}}$ is the identity matrix and hence ${{\mathbf {Q}}}^{(a)} = {{\mathbf {Q}}}^{(b)}$. Since ${{\mathbf {B}}}$ is the identity matrix, we conclude from Theorem 4 that ${{\mathbf {L}}}^{(b)} = {{\mathbf {L}}}^{(a)}$ and we are done. $\square$

#### Example 5

(*An example of non-uniqueness)*Lindqvist and Kjølen (2018) considered the following situation. Let $m=K=2$ and consider the two Coxian models with ${{\mathbf {p}}}^{(a)}={{\mathbf {p}}}^{(b)}=(1,0)'$ and, respectively,

20 $$\begin{aligned} {{\mathbf {Q}}}^{(a)} = \left( \begin{array}{rrr} -4 &{} \quad ~ &{} 1 \\ 0 &{} \quad ~ &{} -5 \end{array} \right) , \; \; {{\mathbf {L}}}^{(a)} = \left( \begin{array}{ccc} 2 &{} \quad ~ &{} \quad 1 \\ 3 &{} \quad ~ &{} \quad 2 \end{array} \right) \end{aligned}$$

and

21 $$\begin{aligned} {{\mathbf {Q}}}^{(b)} = \left( \begin{array}{rrr} -5 &{} \quad ~ &{} \quad 2 \\ 0 &{} \quad ~ &{} \quad -4 \end{array} \right) , \; \; {{\mathbf {L}}}^{(b)} = \left( \begin{array}{ccc} 2 &{} \quad ~ &{} \quad 1 \\ 5/2 &{} \quad ~ &{} \quad 3/2 \end{array} \right) \end{aligned}$$

It was shown that these different representations lead to the same subdensities, namely

22 $$\begin{aligned} f_1(t)= & {} 5e^{-4t} - 3e^{-5t} \end{aligned}$$

23 $$\begin{aligned} f_2(t)= & {} 3e^{-4t} - 2e^{-5t} \end{aligned}$$

Since ${{\mathbf {Q}}}^{(a)} \ne {{\mathbf {Q}}}^{(b)}$ this shows that the condition of equally ordered diagonals in Theorem 5 cannot be removed. Note also that Theorem 4 can be used to show that the two representations of (20) and (21) give rise to the same competing risks model, using the matrix

$$\begin{aligned} {{\mathbf {B}}}= \left( \begin{array}{rcc} 1 &{} \quad ~ &{} \quad 0 \\ -1 &{} \quad ~ &{} \quad 2 \end{array} \right) . \end{aligned}$$

### Canonical representation of Coxian competing risks distributions

The recent article by Rizk et al. (2021) motivates an extension of the canonical model $({{\tilde{{{\mathbf {p}}}}}},{{\tilde{{{\mathbf {Q}}}}}})$ of Sect. 2.3 to the case of multiple absorbing states. These authors model an emergency department with patients moving through a series of service stations, where each station is modeled by a Coxian phase-type distribution. Then in order to model the movement between stations, they include an additional absorbing state in each station. Hence one absorbing state represents patients leaving the hospital, while the other represents movement to the next station. Their clue is to model the case with two absorbing states using a mixture of two series models like the one considered in Fig. 2. As they note, this approach facilitates statistical inference and the inclusion of covariates. For example, matrix exponentials can then be avoided in the likelihood function.

In the following we use their idea to extend the canonical model of Sect. 2.3 to involve an arbitrary number *K* of absorbing states, thus obtaining a canonical model for the Coxian competing risks situation. We also demonstrate below that properties of the single absorbing state case (Sect. 2.3) imply that this is a valid representation for any competing risks representation $({{\mathbf {p}}},{{\mathbf {Q}}},{{\mathbf {L}}})$ with upper triangular ${{\mathbf {Q}}}$.

**Fig. 4:**
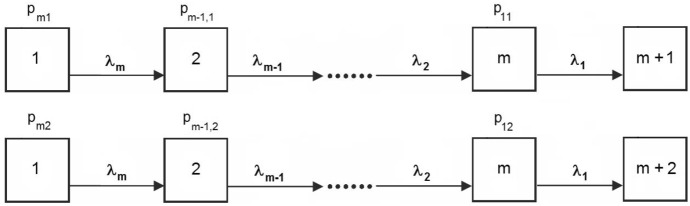
The two possible paths of the canonical representation for Coxian competing risks models with $K=2$ absorbing states. Here $\lambda _1 \ge \lambda _2 \ge \ldots \ge \lambda _m$ and $\sum _{i,j}p_{ij}=1$

As indicated above, the idea is essentially to involve one canonical model of the form $({{\tilde{{{\mathbf {p}}}}}},{{\tilde{{{\mathbf {Q}}}}}})$ (or see Fig. 2) for each absorbing state, and then consider a mixture of them to represent the full competing risks situation. This is illustrated in Fig. 4 for the case $K=2$. Let $p_{ij}$ be the probability of entering the system in state $m-i+1$ $(i=1,2,\ldots ,m)$ and being absorbed in state $m+j$ ($j=1,2,\ldots ,K$). Once entered, the transition rates leading to the absorbing states are identical for each subchain. The parameters of the model are now the $p_{ij}$, which sum to 1 and hence contribute $Km-1$ parameters, in addition to the *m* transition rates $\lambda _i$. Altogether, this gives $(K+1)m-1$ parameters.

In a similar way as for the single absorbing state case, the Laplace transforms of the subdensities $f_j(t)$ of the above representation, can be given in the form [recall (12)]

24 $$\begin{aligned} f_j^*(s) = \sum _{i=1}^m p_{ij} g^*_{(i)}(s) \end{aligned}$$

where the $g^*_{(i)}(s)$ are as given by (13).

Consider then an arbitrary representation $({{\mathbf {p}}},{{\mathbf {Q}}},{{\mathbf {L}}})$ where ${{\mathbf {Q}}}$ is upper triangular. The corresponding Laplace transforms, defined in (19), are of the form

$$\begin{aligned} {{\hat{f}}}_j^*(s) = \frac{{{\hat{N}}}_j(s)}{{{\hat{D}}}(s)} \end{aligned}$$

with ${{\hat{D}}}(s) = \prod _{i=1}^m(s+\lambda _i)$. O’Cinneide (1989) pointed out that the canonical representation $({{\tilde{{{\mathbf {p}}}}}},\tilde{{\mathbf {Q}}})$ of Cumani (1982) also holds for subdensities (actually, O’Cinneide in his papers has included the possibility of a positive probability for $T=0$). This proves that there are nonnegative $p_{ij}$ such that

$$\begin{aligned} {{\hat{f}}}_j^*(s) = \sum _{i=1}^m p_{ij} g^*_{(i)}(s), \end{aligned}$$

which by (24) implies that the representation presented above is equivalent to the $({{\mathbf {p}}},{{\mathbf {Q}}},{{\mathbf {L}}})$. Indeed, the nonnegativeness of the $p_{ij}$ is guaranteed by the result by Cumani (1982) and is a consequence of requiring $\lambda _1 \ge \lambda _2 \ge \ldots \ge \lambda _m$. That the $p_{ij}$ sum to 1, follows since $\sum _{j=1}^K {{\hat{f}}}_j^*(s)$ is the Laplace transform for the absorption time *T*, which is given in (12). Validity and uniqueness of the new representation can now be proven in the same manner as for the single absorbing state case as shown in Cumani (1982). Note the requirement that $\lambda _1 \ge \lambda _2 \ge \ldots \ge \lambda _m$.

In a way similar to the single absorbing state case, an equivalent version of the above canonical model can be given in the form of a Coxian competing risks model with ${{\mathbf {Q}}}$ as given in (10) and $\lambda _1 \ge \lambda _2 \ge \ldots \ge \lambda _m$. Rizk et al. (2021) present formulas for going from the above canonical representation to the Coxian model representation for the case $K=2$.

*Example 5 (cont.)* We show how to derive the canonical representation of the competing risks model considered in the first part of Example 5. First, calculate the Laplace transforms corresponding to $f_1(t)$ and $f_2(t)$ in (22) and (23),

$$\begin{aligned} f^*_1(s)= & {} \frac{5}{s+4}-\frac{3}{s+5} = \frac{2s + 13}{(s+4)(s+5)} \\ f^*_2(s)= & {} \frac{3}{s+4}-\frac{2}{s+5} = \frac{s+7}{(s+4)(s+5)} \end{aligned}$$

With notation as above, we further get by letting $\lambda _1=5, \lambda _2=4$,

$$\begin{aligned} g^*_{(1)}(s)= & {} \frac{\lambda _1}{s+\lambda _1} = \frac{5}{s+5} \\ g^*_{(2)}(s)= & {} \frac{\lambda _1 \lambda _2}{(s+\lambda _1)(s+\lambda _2)} = \frac{20}{(s+5)(s+4)} \end{aligned}$$

By equating coefficients on each side of (24) with the above functions, we arrive at

$$\begin{aligned} p_{11} = 0.40, \; p_{21} = 0.25, \;p_{12} = 0.20, \;p_{22} = 0.15. \end{aligned}$$

The alternative canonical Coxian model turns out to be simply $({{\mathbf {Q}}}^{(b)},{{\mathbf {L}}}^{(b)})$, given in the beginning of Example 5.

### Maximum likelihood estimation in the canonical model

As noted by Rizk et al. (2021), the representation for Coxian competing risks models given in the previous subsection, turns out to be very well suited for statistical inference. We consider below for simplicity the case of right-censored competing risks data without covariates, while a note on the inclusion of covariates is given in the end, following the suggestion of Rizk et al. (2021).

Let the observed survival time for individual *k* ($k=1,2,\ldots ,N$, say) be $T_k$ and let $D_k$ be the corresponding status variable, where

$$\begin{aligned} D_k = \left\{ \begin{array}{cl} 0 &{} \quad \text{ if }\ T_k\ \text{ is } \text{ a } \text{(right) } \text{ censoring } \text{ time } \\ j &{} \quad \text{ if }\ T_k\ \text{ is } \text{ the } \text{ time } \text{ of } \text{ absorption } \text{ in } \text{ state }\ m+j, j=1,2,\ldots ,K \end{array} \right. . \end{aligned}$$

Consider the modeling of these data using the model of Sect. 3.4. The parameters are hence $p_{ij}$ for $i=1,\ldots ,m; \;j=1,2,\ldots ,K$, noting that $p_{mK} = 1-\sum _{(i,j) \ne (m,K)}p_{ij}$, and $\lambda _1,\ldots ,\lambda _m$. For the latter parameters, we shall assume strict inequalities in $\lambda _1> \ldots > \lambda _m$, which simplifies the likelihood and which seems reasonable when there is no apriori modeled connection between the $\lambda _i$.

Several authors have suggested using the EM-algorithm for maximum likelihood estimaton in phase-type models (see, e.g., Asmussen et al. 1996). The clue is then to let the states visited by the Markov chain on the way to absorption, define the latent observations. This simplifies the full likelihood and its maximization in the M-step, while the E-step is usually also rather straightforward to perform. As we shall see, the canonical representation considered here allows a very simple and transparent use of the EM-algorithm.

Let then the (latent) starting state for individual *k* be defined by the vector

$$\begin{aligned} {{\mathbf {X}}}_k = (X_{k,ij}; i=1,\ldots ,m, \; j=1,\ldots ,K), \end{aligned}$$

where $X_{k,ij} = 1$ if the *k*th individual starts in state $m-i+1$ in the *j*th subchain, while the other entries in ${{\mathbf {X}}}_k$ equal 0.

The likelihood contribution for an item that starts in state $m-i+1$ with $i \in \{1,2,\ldots ,m\}$ and is absorbed in state $m+j$ for $j \in \{1,2,\ldots ,K\}$ after a time *t* is

$$\begin{aligned} p_{ij} g_{(i)}(t;{\varvec{\lambda }}), \end{aligned}$$

where $p_{ij}$ is defined in Sect. 3.4, $g_{(i)}(t;{\varvec{\lambda }})$ is the density of $U_1+\ldots +U_i$ when $U_{\ell }$ for $\ell =1,2,\ldots ,m$ is the waiting time in state $\ell$, which is exponentially distributed with rate $\lambda _\ell$, and ${\varvec{\lambda }}=(\lambda _1,\ldots ,\lambda _m)$. The density $g_{(i)}$ is given by

$$\begin{aligned} g_{(i)}(t;{\varvec{\lambda }}) = \sum _{\ell =1}^i\frac{\prod _{u=1}^i \lambda _u}{\prod _{u \ne \ell }^i (\lambda _u-\lambda _\ell )}e^{-\lambda _\ell t}. \end{aligned}$$

For right censored observations, the likelihood contribution for an item that starts in state $m-i+1$ in the *j*th subchain, and is right censored at time *t*, is

$$\begin{aligned} p_{ij} S_{(i)}(t;{\varvec{\lambda }}). \end{aligned}$$

Here, $S_{(i)}(t;{\varvec{\lambda }})$ is the survival function of $U_1+\ldots +U_i$, given by

$$\begin{aligned} S_{(i)}(t;{\varvec{\lambda }}) = \sum _{\ell =1}^i\frac{\prod _{u=1,u\ne \ell }^i \lambda _u}{\prod _{u \ne \ell }^i (\lambda _u-\lambda _\ell )}e^{-\lambda _\ell t}. \end{aligned}$$

The contribution to the log-likelihood function from a single individual *k* can then be written

$$\begin{aligned} L_k= & {} \sum _{j=1}^K \sum _{i=1}^m X_{k,ij}I(D_k=j)(\log (p_{ij})+\log (g_{(i)}(T_k;{\varvec{\lambda }})))\\+ & {} \sum _{j=1}^K \sum _{i=1}^m X_{k,ij}I(D_k=0)(\log (p_{ij})+\log (S_{(i)}(T_k;{\varvec{\lambda }}))), \end{aligned}$$

so the full log-likelihood function for the data plus latent variables is $L = \sum _{k=1}^N L_k$.

For the E-step of the algorithm we get for $j=1,\ldots ,K$,

$$\begin{aligned} E(X_{k,ij}|D_k=j,T_k=t)= \frac{g_{(j)}(t;{\varvec{\lambda }})p_{ij}}{\sum _{\ell =1}^m g_{(\ell )}(t;{\varvec{\lambda }})p_{\ell j}} \end{aligned}$$

and

$$\begin{aligned} E(X_{k,ij}|D_k=0,T_k=t)= \frac{S_{(j)}(t;{\varvec{\lambda }})p_{ij}}{\sum _{\ell =1}^m \sum _{r=1}^K S_{(\ell )}(t;{\varvec{\lambda }})p_{\ell r}}. \end{aligned}$$

The M-step is a maximization of the log-likelihood function *L* with respect to the parameters $p_{ij}$ and ${\varvec{\lambda }}$, with the restriction $\lambda _1> \lambda _2 > \ldots \lambda _m$.

Following Rizk et al. (2021), if ${{\mathbf {z}}}_k$ is a covariate vector for individual *k*, then we may let the covariates influence the rates $\lambda _i$ in the way $\lambda _i \exp \{{\varvec{\beta }}'{{\mathbf {z}}}_k\}$. In particular, this will maintain the inequalities between the $\lambda$-parameters for each individual.

#### Example 6

(Real data case) As an illustration we fitted competing risks data from Beyersmann et al. (2012, Ch. 1). The data are observations from an intensive care unit, with the purpose to examine the effect of hospital-acquired infections. We analyzed the data for patients without pneumonia at admission to the unit. The time variable was length of stay at the intensive care unit, with two outcomes of interest, either *alive discharge* ($D=1$) or *hospital death* ($D=2$). There were 650 patients in these data, with 589 being discharged alive, 55 dead in hospital, and 6 right censored.

Using the model of Fig. 4 with $m=3$, we obtained the estimates ${{\hat{p}}}_{31}=0.8712$, ${{\hat{p}}}_{21}=0.0410$, ${{\hat{p}}}_{32}=0.0878$, ${{\hat{p}}}_{11}={{\hat{p}}}_{22}={{\hat{p}}}_{12}=0.0000$, $\hat{\lambda }_1 = 1.5147$, ${{\hat{\lambda }}}_2 = 0.6938$, ${{\hat{\lambda }}}_3 = 0.0946$.

**Fig. 5:**
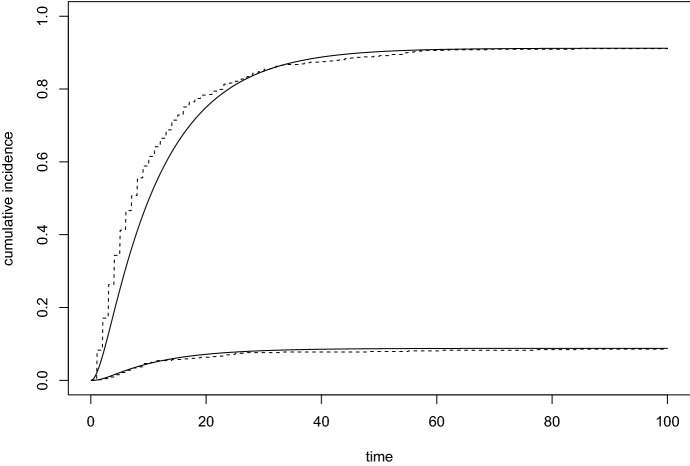
Estimated cumulative incidence functions for the data example. The upper pair of curves is for the outcome ‘alive discharge’ and the lower pair is for ‘hospital death’. Solid curves are estimates from the phase-type model; dashed curves are estimates using the nonparametric Aalen–Johansen estimator

Figure 5 shows the estimated cumulative incidence functions obtained from the phase-type model, together with nonparametric estimates found by using the Aalen–Johansen estimators (see, e.g., Borgan 1998). With the nonparametric curve serving as a “benchmark”, the fit seems very good for the outcome ‘hospital death’, while the model with $m=3$ is seemingly not able to pick up completely the steepness of the first part of the cumulative incidence function for the outcome ‘alive discharge’. A seemingly better fit was obtained, on the other hand, using a model with $m=4$ in Lindqvist and Kjølen (2018).

## Concluding remarks

In this paper we have studied the concept of phase-type distributions and how they can can be extended to cover the modeling of competing risks in survival analysis. For a proper treatment of the competing risks case it has been necessary to review some basic parts of the theory of ordinary phase-type distributions, and in particular the so-called Coxian phase-type distributions.

As already indicated in the Introduction, there appears currently to be a considerable research activity in the field of phase-type distributions. Indeed, the increased opportunity of handling large data sets using complicated models, has led to an interest in the use of phase-type models for example in health studies. One of the first papers of this kind was Faddy et al. (2009) who claimed the superiority of Coxian phase-type models over common parametric models like gamma and lognormal.

The new applications have also motivated new theoretical developments and extensions of the classical models. For example, while the present study has been limited to phase-type modeling via homogeneous Markov chains, there have recently been published papers which involve nonhomogenous Markov chains (Bladt and Yslas 2020), semi-Markov models (Garcia-Maya et al. 2021), and models including unobserved heterogeneity (Surya 2016). Guihenneuc-Jouyaux et al. (2000) considered a Markov model of disease progression with a single absorbing state, where noisy biomarker measurements, depending on the state of the Markov chain, were available.

The main emphasis of the present paper has been on identifiability of phase-type models and their extensions to competing risks, with motivation from statistical inference. A basic problem has been to characterize representations $({{\mathbf {p}}}^{(a)},{{\mathbf {Q}}}^{(a)})$ and $({{\mathbf {p}}}^{(b)},{{\mathbf {Q}}}^{(b)})$ which lead to the same phase-type distribution. Most of the discussion has involved representations of the same dimension, which perhaps is of most interest in practical statistical modeling. The case of upper triangular matrices ${{\mathbf {Q}}}$, where phase-type distributions can be represented by simple canonical versions, is seemingly well understood, see Cumani (1982) and the more recent paper by He and Zhang (2006). On the other hand, the general case, which may include *complex* eigenvalues (poles), seems to have less established general results. For example, it is not known whether there exist a kind of canonical representation for such cases (see, e.g., O’Cinneide 1999).

Some remarkable, but seemingly not much developed, results for equivalence of representations $({{\mathbf {p}}}^{(a)},{{\mathbf {Q}}}^{(a)})$ and $({{\mathbf {p}}}^{(b)},{{\mathbf {Q}}}^{(b)})$ of different dimensions were given by Ryden (1996). The results were obtained by specializing more general results from Ito et al. (1992) for discrete time Markov chains. The underlying idea was that a phase-type distribution can be represented as an *aggregated* Markov chain, where there are two compartments in the aggregated process, $\{1,2,\ldots ,m\}$ and $\{m+1\}$. There is hence a natural extension to the competing risks case. A further study in this direction will certainly be of interest.

## Appendix

### A1. Matrix functions

Let ${{\mathbf {A}}}$ be a $m \times m$ matrix. Let the distinct eigenvalues of ${{\mathbf {A}}}$ be $\rho _k$ for $k=1,2,\ldots ,v$, and let their multiplicities of the *minimal* polynomial (Horn and Johnson 2013, Ch. 3.3) be, respectively, $m_k$. Then for an analytic function *f* we can write (Dunford and Schwartz 1958, Theorem 8, Chapter VII)

25 $$\begin{aligned} f({{\mathbf {A}}}) = \sum _{k=1}^v \sum _{r=0}^{m_k-1} \frac{f^{(r)}(\rho _k)}{r!} ({{\mathbf {A}}}-\rho _k {{\mathbf {I}}})^r {{\mathbf {Z}}}_{k} \end{aligned}$$

where the matrices ${{\mathbf {Z}}}_{k}$ are polynomials in ${{\mathbf {A}}}$ which do not depend on *f* and furthermore satisfy

- $${{\mathbf {Z}}}_k \ne {{\mathbf {0}}}$$
- $${{\mathbf {Z}}}_k^2 = {{\mathbf {Z}}}_k \text{ and } {{\mathbf {Z}}}_k{{\mathbf {Z}}}_\ell = {{\mathbf {0}}} \text{ for } k \ne \ell $$
- $$\sum _{k=1}^v {{\mathbf {Z}}}_k = {{\mathbf {I}}}$$
- $$({{\mathbf {A}}}-\rho _k {{\mathbf {I}}})^{m_k} {{\mathbf {Z}}}_k = {{\mathbf {0}}}$$

### A2. More on nonredundancy of $({{\mathbf {p}}},{{\mathbf {Q}}})$ and its consequences

Referring to Sect. 2.1, we may express the Laplace transform (6) using (25), obtaining

26 $$\begin{aligned} f^*(s)= 1 - {{\mathbf {p}}}' \left\{ \sum _{k=1}^v \sum _{r=0}^{m_k-1} s(s-\rho _k)^{-r-1}({{\mathbf {Q}}}-\rho _k{{\mathbf {I}}})^r {{\mathbf {Z}}}_k \right\} {{\mathbf {1}}}. \end{aligned}$$

Writing this using a common denominator, the denominator will be of the form

$$\begin{aligned} \prod _{k=1}^v (s-\rho _k)^{m_k}, \end{aligned}$$

which is a polynomial of degree $\sum _{k=1}^v m_k \le m$. Thus nonredundacy of $({{\mathbf {p}}},{{\mathbf {Q}}})$, as defined in Sect. 2.2, requires that the $m_k$ sum to m, which means that the *minimal* polynomial equals the *characteristic* polynomial.

It next follows that if $({{\mathbf {p}}},{{\mathbf {Q}}})$ is nonredundant, then

27 $$\begin{aligned} {{\mathbf {p}}}'({{\mathbf {Q}}}-\rho _k {{\mathbf {I}}})^{m_k-1} {{\mathbf {Z}}}_{k}{{\mathbf {1}}}\ne 0 \end{aligned}$$

for $k=1,2,\ldots ,v$. This is seen from (26), since the definition of nonredundancy implies that the term $s(s-\rho _k)^{-m_k}$ must have a nonzero coefficient.

Now suppose that $({{\mathbf {p}}}^{(a)},{{\mathbf {Q}}}^{(a)})$ and $({{\mathbf {p}}}^{(b)},{{\mathbf {Q}}}^{(b)})$ represent the same phase-type distribution. Then by (4) we have

$$\begin{aligned} {{{\mathbf {p}}}^{(a)}}'e^{{{\mathbf {Q}}}^{(a)}t} {{\mathbf {1}}}= {{{\mathbf {p}}}^{(b)}}'e^{{{\mathbf {Q}}}^{(b)}t} {{\mathbf {1}}}\end{aligned}$$

Since for any ${{\mathbf {p}}}$ and ${{\mathbf {Q}}}$, by (25),

28 $$\begin{aligned} {{\mathbf {p}}}'e^{{{\mathbf {Q}}}t} {{\mathbf {1}}}= \sum _{k=1}^v \sum _{r=0}^{m_k-1} \frac{t^r e^{\rho _kt}}{r!}{{\mathbf {p}}}' ({{\mathbf {Q}}}-\rho _k {{\mathbf {I}}})^r {{\mathbf {Z}}}_{k} {{\mathbf {1}}}, \; \; \; \; \end{aligned}$$

it follows from (27) that ${{\mathbf {Q}}}^{(a)}$ and ${{\mathbf {Q}}}^{(b)}$ must have the same eigenvalues with the same multiplicities. Moreover, (27) together with $\sum _k m_k=m$ implies that ${{\mathbf {Q}}}^{(a)}$ and ${{\mathbf {Q}}}^{(b)}$ must have the same Jordan block representation (Horn and Johnson (2013), Ch.3.1), where there is exactly one Jordan block for each eigenvalue. This implies in turn that ${{\mathbf {Q}}}^{(a)}$ and ${{\mathbf {Q}}}^{(b)}$ are similar in the sense that there is an invertible matrix ${{\mathbf {B}}}$ such that

$$\begin{aligned} {{\mathbf {Q}}}^{(b)}= {{\mathbf {B}}}^{-1}{{\mathbf {Q}}}^{(a)}{{\mathbf {B}}}, \end{aligned}$$

see the proof of Theorem 1 in Telek and Horváth (2007). Furthermore, the above property of the Jordan representation is shown by Telek and Horváth (2007) to imply that ${{\mathbf {B}}}$ can be chosen so that ${{\mathbf {B}}}{{\mathbf {1}}}={{\mathbf {1}}}$.

O’Cinneide (1989) introduced the notion of a *simple* phase-type generator ${{\mathbf {Q}}}$. Let $P\!H({{\mathbf {Q}}})$ be the set of all phase-type distributions with representation $({{\mathbf {p}}},{{\mathbf {Q}}})$ for *some* ${{\mathbf {p}}}$. Then ${{\mathbf {Q}}}$ is called *simple* if each distribution in $P\!H({{\mathbf {Q}}})$ has a unique representation $({{\mathbf {p}}},{{\mathbf {Q}}})$. It can be shown (He and Zhang 2006) that ${{\mathbf {Q}}}$ is simple if and only if the minimal polynomial of ${{\mathbf {Q}}}$ equals the characteristic polynomial. Thus, nonredundancy of $({{\mathbf {p}}},{{\mathbf {Q}}})$ implies that ${{\mathbf {Q}}}$ is simple.

O’Cinneide (1989) showed that for two simple matrices ${{\mathbf {Q}}}^{(a)}$ and ${{\mathbf {Q}}}^{(b)}$ of the same dimension, $P\!H({{\mathbf {Q}}}^{(a)}) = P\!H({{\mathbf {Q}}}^{(b)})$ if and only if there is a permutation matrix ${{\mathbf {U}}}$ such that ${{\mathbf {Q}}}^{(a)}{{\mathbf {U}}}= {{\mathbf {U}}}{{\mathbf {Q}}}^{(b)}$. This condition is considerably stronger than the one of Telek and Horváth (2007) provided in our Theorem 1, where however the latter is concerned with comparison of two *single* distributions instead of two *sets* of distributions.

### A3. Proof of Theorem 4

Suppose $F_j^{(a)}(t)= F_j^{(b)}(t)$ for all $t>0$. Then the corresponding cumulative distribution functions of *T* satisfy

$$\begin{aligned} F^{(a)}(t)= \sum _{j=1}^K F_j^{(a)}(t) = \sum _{j=1}^K F_j^{(b)}(t)= F^{(b)}(t). \end{aligned}$$

Theorem 1 then implies that there is an $m \times m$ invertible matrix ${{\mathbf {B}}}$ such that

$$\begin{aligned} {{\mathbf {B}}}{{\mathbf {1}}}= & {} {{\mathbf {1}}}\\ {{{\mathbf {p}}}^{(b)}}'= & {} {{{\mathbf {p}}}^{(a)}}' {{\mathbf {B}}}\\ {{\mathbf {Q}}}^{(b)}= & {} {{\mathbf {B}}}^{-1}{{\mathbf {Q}}}^{(a)} {{\mathbf {B}}}\end{aligned}$$

It follows by (17) that

29 $$\begin{aligned} f_j^{(b)}(t)= & {} {{{\mathbf {p}}}^{(b)}}'e^{{{\mathbf {Q}}}^{(b)} t}{\varvec{\ell }}_j^{(b)} \nonumber \\= & {} {{{\mathbf {p}}}^{(a)}}' {{\mathbf {B}}}{{\mathbf {B}}}^{-1} e^{{{\mathbf {Q}}}^{(a)} t}{{\mathbf {B}}}{\varvec{\ell }}_j^{(b)} \nonumber \\= & {} {{{\mathbf {p}}}^{(a)}}' e^{{{\mathbf {Q}}}^{(a)} t} {{\mathbf {B}}}{\varvec{\ell }}_j^{(b)} \nonumber \\= & {} {{{\mathbf {p}}}^{(a)}}' e^{{{\mathbf {Q}}}^{(a)} t} {\varvec{\ell }}_j^{(a)} \end{aligned}$$

where the last equality follows since, by the assumption of the theorem, $f_j^{(b)}(t) = f_j^{(a)}(t)$. The question is therefore whether the above implies ${{\mathbf {B}}}{\varvec{\ell }}_j^{(b)}= {\varvec{\ell }}_j^{(a)}$ for each *j*, or equivalently, whether ${{\mathbf {B}}}{{\mathbf {L}}}^{(b)}= {{\mathbf {L}}}^{(a)}$.

We are done if we can show that, for any nonredundant representation $({{\mathbf {p}}},{{\mathbf {Q}}})$ and *m*-vector ${{\mathbf {g}}}$,

$$\begin{aligned} {{\mathbf {p}}}' e^{{{\mathbf {Q}}}t}{{\mathbf {g}}}= 0 \text{ for } \text{ all }\ t>0 \end{aligned}$$

implies ${{\mathbf {g}}}={{\mathbf {0}}}$. Thus assume

30 $$\begin{aligned} {{\mathbf {p}}}'e^{{{\mathbf {Q}}}t} {{\mathbf {g}}}= \sum _{k=1}^v \sum _{r=0}^{m_k-1} \frac{t^r e^{\rho _kt}}{r!}{{\mathbf {p}}}' ({{\mathbf {Q}}}-\rho _k {{\mathbf {I}}})^r {{\mathbf {Z}}}_{k} {{\mathbf {g}}}\; = \; 0 \; \text{ for } \text{ all } t>0, \; \; \; \; \end{aligned}$$

By linear independence of the involved functions of *t* it follows that

31 $$\begin{aligned} {{\mathbf {p}}}' ({{\mathbf {Q}}}-\rho _k {{\mathbf {I}}})^r {{\mathbf {Z}}}_{k} {{\mathbf {g}}}\; = \; 0 \; \text{ for } k=1,\ldots ,v; \; r=0,\ldots ,m_k-1. \end{aligned}$$

This amounts to *m* conditions for ${{\mathbf {g}}}$, each involving an *m*-vector

$$\begin{aligned} {{\mathbf {c}}}_{kr}' = {{\mathbf {p}}}' ({{\mathbf {Q}}}-\rho _k {{\mathbf {I}}})^r {{\mathbf {Z}}}_{k}. \end{aligned}$$

We now show that ${{\mathbf {c}}}_{kr} \ne {{\mathbf {0}}}$ for each *k*, *r*. This follows from (27). More precisely, (27) first implies that ${{\mathbf {p}}}' ({{\mathbf {Q}}}-\rho _k {{\mathbf {I}}})^{m_k-1} {{\mathbf {Z}}}_{k} \ne {{\mathbf {0}}}$, and then by using that ${{\mathbf {Z}}}_k$ commutes with $({{\mathbf {Q}}}-\rho _k {{\mathbf {I}}})^{m_k-1}$ (itself being a polynomial in ${{\mathbf {Q}}}$), it follows that ${{\mathbf {p}}}' ({{\mathbf {Q}}}-\rho _k {{\mathbf {I}}})^{r} {{\mathbf {Z}}}_{k} \ne {{\mathbf {0}}}$ for $r=0,1,\ldots ,m_k-1$.

Now let ${{\mathbf {C}}}$ be the $m \times m$ matrix with rows all the ${{\mathbf {c}}}'_{kr}$. Then (31) is simply

$$\begin{aligned} {{\mathbf {C}}}{{\mathbf {g}}}= {{\mathbf {0}}}\end{aligned}$$

and we are done if we can show that ${{\mathbf {C}}}$ is invertible. We do this by showing that the vectors ${{\mathbf {c}}}'_{kr}$ are linearly independent. Thus assume that

$$\begin{aligned} \sum _{k=1}^v \sum _{r=0}^{m_k-1} \gamma _{kr} {{\mathbf {p}}}' ({{\mathbf {Q}}}-\rho _k {{\mathbf {I}}})^r {{\mathbf {Z}}}_{k} = {{\mathbf {0}}}. \end{aligned}$$

Multiplying from the right by ${{\mathbf {Z}}}_{\ell }$ for $\ell \in \{1,2,\ldots ,v\}$ and using properties (a)–(b) of Appendix A1 we get

32 $$\begin{aligned} \sum _{r=0}^{m_{\ell }-1} \gamma _{\ell r} {{\mathbf {p}}}' ({{\mathbf {Q}}}-\rho _{\ell } {{\mathbf {I}}})^r {{\mathbf {Z}}}_{\ell } = {{\mathbf {0}}}\end{aligned}$$

Multiplying this from the right by $({{\mathbf {Q}}}-\rho _{\ell } {{\mathbf {I}}})^{m_{\ell }-1}$ and using (d) of “Appendix A1” with ${{\mathbf {A}}}={{\mathbf {Q}}}$, we get

$$\begin{aligned} \gamma _{\ell 0} {{\mathbf {p}}}' ({{\mathbf {Q}}}-\rho _{\ell } {{\mathbf {I}}})^{m_{\ell }-1} {{\mathbf {Z}}}_{\ell } \equiv \gamma _{\ell 0} {{\mathbf {c}}}'_{\ell ,m_{\ell }-1} = {{\mathbf {0}}}\end{aligned}$$

and hence $\gamma _{\ell 0} = 0$ since ${{\mathbf {c}}}'_{\ell ,m_{\ell }-1} \ne {{\mathbf {0}}}$. Putting $\gamma _{\ell 0} = 0$ in () and multiplying from the right by $({{\mathbf {Q}}}-\rho _{\ell } {{\mathbf {I}}})^{m_{\ell }-2}$, we get $\gamma _{\ell 1} = 0$. Continuing in this way we prove that $\gamma _{\ell r} = 0$ for $r=0,1,\ldots ,m_{\ell }-1$. Then this can be done for any $\ell =1,2,\ldots ,v$ and the necessity part of Theorem 4 is proven. To prove sufficiency is, on the other hand, straightforward.
